# Relationship between allergic rhinitis and allergic conjunctivitis (allergic rhinoconjunctivitis) - review

**DOI:** 10.22336/rjo.2022.3

**Published:** 2022

**Authors:** Andreea Iordache, Mădălina Boruga, Ovidiu Mușat, Daniel Alexandru Jipa, Călin Petru Tătaru, Gabriela Cornelia Mușat

**Affiliations:** * “Victor Babeș”, University of Medicine and Pharmacy Timișoara, Romania; ** “Carol Davila” Central Military Emergency Hospital, Bucharest, Romania; *** “Pius Brînzeu” County Hospital, Timișoara, Romania; **** “Carol Davila” University of Medicine and Pharmacy, Bucharest, Romania; ***** Clinical Ophthalmology Emergency Hospital, Bucharest, Romania; ****** “Sf. Maria” Clinical Hospital, Bucharest, Romania

**Keywords:** allergic rhinitis, allergic conjunctivitis, allergic rhinoconjunctivitis

## Abstract

Allergic rhinitis and allergic conjunctivitis are so frequently associated that the need to coin a new name to describe the simultaneous manifestations generated the term allergic rhinoconjunctivitis. The significant impact of rhinoconjunctivitis on the quality of life and the wellbeing of the patients is the reason why the medical community shows a great interest to this disease. Another aspect is the financial burden that is not negligible. The anatomical connection between the organs involved facilitates the propagation of the disease. The allergic pathophysiological mechanisms implicated in allergic rhinitis and conjunctivitis also share common features. The diagnosis of rhinoconjunctivitis is based on the concordance between the symptoms, the clinical examination, and the diagnostic tests that should reveal the existence of an allergen specific IgE in vivo or in vitro. Whilst the nasal smear for eosinophils is considered a reliable diagnostic test for allergic rhinitis, the occurrence of eosinophils in the conjunctive is not a trustworthy indicator of allergy. The therapy of allergic rhinoconjunctivitis is based on patient education, pharmacotherapy, and allergen-specific immunotherapy. The local treatment for the allergic rhinitis is primarily based on topical corticosteroids that also manage the ocular symptoms. The first line of treatment of the ocular manifestations is represented by topical antihistamines and mast-cell stabilizers or double action drugs.

## Introduction

The patients with allergic rhinitis and other respiratory allergies are commonly affected by symptoms of ocular allergy. Allergic rhinitis and allergic conjunctivitis are often concomitant diseases showing a strong epidemiological correlation. Allergic rhinitis and allergic conjunctivitis are so frequently associated that the need to coin a new name to describe the simultaneous manifestations generated the term allergic rhinoconjunctivitis. 

Allergic rhinitis cannot be regarded as an isolated pathology. There are other allergic disorders that are usually associated. Whenever there is an allergic inflammation of the nasal fossae, this inflammation will also involve the mucosa of the sinuses, the mucosa of the middle ear, and the conjunctiva of the eye [**1**].

Although both conditions, allergic rhinitis, and conjunctivitis, may be regarded as trivial, their impact on the general wellbeing and the quality of life of the patients is significant [**2**,**3**]. The financial burden of the disease is not negligible since patients with allergic rhinitis have a twofold increase in medication cost compared to non-allergic patients. In US, allergic rhinitis is the cause for 3.5 million lost workdays and 2 million lost schooldays annually [**4**,**5**].

## Definitions, terminology, classifications 

Allergic rhinitis is an inflammatory IgE mediated disease of the nasal mucosa characterized by nasal congestion, rhinorrhea, sneezing and/ or nasal itching. It can also be defined as an inflammation of the nasal mucosa occurring when a person inhales an allergen. Allergic rhinitis is categorized in connection with the temporal pattern of exposure to triggering agents, to the severity of the symptoms or the frequency of the symptoms. Traditionally, allergic rhinitis is classified into seasonal or perennial allergic rhinitis. The classification in connection with the severity and the frequency of symptoms allows a more appropriate treatment selection. This classification divides allergic rhinitis into intermittent (< 4 days/ week or < 4 weeks/ year) and persistent (> 4 days/ week or > 4 weeks/ year) [**6**]. In relation with the severity of the symptoms, allergic rhinitis can be divided into mild or severe. 

Allergic conjunctivitis is defined as an allergic inflammation of the conjunctiva mediated by IgE. The most common allergic reaction of the eye is type I hypersensitivity reaction of the Gel-Coombs classification. There are several subtypes of allergic conjunctivitis: seasonal allergic, perennial allergic, atopic keratoconjunctivitis, giant papillary conjunctivitis, limbal and tarsal vernal conjunctivitis. 

Seasonal allergic conjunctivitis and perennial allergic conjunctivitis differ by the timeframe of the symptoms. Whilst seasonal allergic conjunctivitis symptoms are mainly manifested for a defined period of time as a response to airborne allergens (i.e., grass, weeds, tree pollen) during the spring, summer or autumn [**7**], the perennial allergic conjunctivitis symptoms last all year round as other types of allergens are involved (dust, mite, molds, pet dander).

Vernal keratoconjunctivitis is an inflammation of the conjunctiva occurring in individuals with an atopic terrain. Conjunctival scarring and corneal complications are commonly associated with vernal keratoconjunctivitis. It is not associated with skin positive testing in 42-47% of cases proving that it is not just a IgE mediated disorder [**8**].

Atopic keratoconjunctivitis is a bilateral inflammation of the conjunctiva and of the eyelids connected with atopic dermatitis. A type I hypersensitivity disorder is involved in the pathophysiology of the disease [**9**]. 

Giant papillary conjunctivitis is a disease of the superior tarsal conjunctiva recognizing an immune mediated mechanism. It is believed that there is a combination between the type I and type IV hypersensitivity responses. M cells and B lymphocytes may be implicated in the pathophysiologic mechanisms of giant papillary conjunctivitis [**10**]. 

## Epidemiology

Allergies represent the fifth leading group in chronic diseases, but the real prevalence of the involvement of ocular allergy is not well determined. A study on a on a substantial population published in 2010 found that up to 40% of the participants experienced ocular symptoms at least once in their lifetime [**11**].

A study with 200 participants on subjects diagnosed with allergic rhinitis stated that approximately 90% of them also had ocular symptomatology [**12**].

Bousquet et al. found that approximately 88% of allergic rhinitis patients showing sensibilization to cypress pollen had concomitant allergic conjunctivitis [**13**]. 

A study from Switzerland on 509 allergic patients found rhinoconjunctivitis in 87,3% of cases, rhinitis without conjunctivitis in 6,7%, and conjunctivitis without rhinitis in 8% of cases. 

Evidence suggested that in seasonal allergic rhinitis, ocular symptoms are more frequent than in perennial allergic rhinitis [**14**].

## Pathophysiology

The original Gel and Coombs classification of allergic reactions divides the immune response in four subtypes. Type I immediate or IgE mediated; type II, cytotoxic or IgM/ IgG mediated; type III IgG/ IgM immune complex mediated; type IV delayed type hypersensitivity or T cell mediated [**15**,**16**].

The sensitivity to allergens is predetermined by a genetic tendency. When exposed to a foreign inhaled protein, predisposed individuals develop a sensitivity reaction that leads to the production of specific IgE against these proteins. When the allergen is inhaled, it binds the specific IgE situated on the exterior of the mast cells generating the release of allergic mediators. The allergic immune response recognizes two stages, the early phase, and the late phase. The mediators involved in the early phase are histamine, tryptase, kinase, kinins, heparin, leukotrienes, and prostaglandins. These mediators account for the symptoms specific to allergic rhinitis: rhinorrhea, sneezing itching, nasal congestion. Mucous glands are stimulated, and the vascular permeability is increased explaining the rhinorrhea; vasodilatation explains the nasal congestion; sensory nerves are stimulated explaining the sneezing and itching. The early phase occurs in the first few minutes after the contact with the allergen. The late-phase response starts 4-8 hours after the exposure and may last for days. This phase is distinguished by the fact that it generates the recruitment of inflammatory cells such as neutrophils, eosinophils, lymphocytes, and macrophages. The symptoms of the late phase are similar to those of the early phase with the difference that the congestion and the mucus production is increased whilst the itching and the sneezing are diminished [**17**,**18**]. 

Household dust and pollen were identified as most likely to induce both nasal and conjunctival symptoms producing rhinoconjunctivitis [**11**].

Unified airways disease is a theory that is built on the consideration that the upper and lower airways are a unified morphologic and functional unit. There are strong clinical, pathophysiological, and epidemiologic evidence that sustain this theory. The mucosa of the nose and of the bronchi present similarities and are constituted of ciliary epithelium, basement membrane, lamina propria, glands, and goblet cells, forming the so-called united airway that explains the same way of reaction. Rhinitis and asthma are chronic inflammatory diseases of the upper and lower respiratory system sharing the same allergic and non-allergic mechanisms. The treatment of rhinitis and asthma must be addressed to both inferior and superior airways in such a way to achieve a management of both diseases [**19**]. 

The conjunctiva is a mucosal surface resembling the nasal mucosa, having the same type of epithelium so it is rational to consider that the reactivity to both allergic and non-allergic aggressors is similar. An allergic inflammation that affects the nasal mucosa will also be present in the eye conjunctiva, the mucosa of the sinuses, the mucosa of the middle ear, and the lower airways. 

The anatomical connection between the organs involved facilitates the propagation of the disease. The allergic pathophysiological mechanisms implicated in allergic rhinitis and conjunctivitis also share common features [**20**]. 

The anatomical connection between the eye and the inferior meatus of the nasal fossae is realized by the nasolacrimal duct. This duct is a pathway through which the allergens and the mediators from the conjunctiva drain along with the tears in the meatus under the inferior turbinate. Nasolacrimal reflux with upward migration has been allegedly noticed by some authors but is considered unlikely to happen. The blockage of the nasolacrimal duct is expected to increase the tearing considering a simple mechanical perspective [**21**]. Treatment of the nasal allergy with intranasal corticosteroid can restore the patency of the nasolacrimal duct and diminish the ocular symptoms [**22**].

Multiple studies have established the fact that there is a nasal-ocular reflex, which explains the interrelation between the nasal and the ocular pathology. The irritation of the nasal mucosa produced by the histamine induces a response mediated by the parasympathetic nervous system. This response is experienced at the level of the conjunctiva and at the level of the opposite nasal cavity, thus generating the symptoms [**23**]. 

Nasal allergy also induces an inflammatory response at a systemic level. This systemic upregulation might be responsible for a more rapid and intense infiltration of inflammatory cells in the conjunctiva [**24**,**25**].

## Histopathology

In acute allergic conjunctivitis, mast cells are increased in number in bulbar and tarsal substantia propria. Eosinophils are present in some cases, but their absence does not eliminate the diagnosis of allergy. A study on the cytologic examination of the tarsal conjunctival scrapping of 4 groups of patients with pollen allergic conjunctivitis, atopy without conjunctivitis, acute nonallergic conjunctivitis, and normal subjects, concluded that the occurrence of eosinophils in the conjunctive is not a reliable indicator of allergy [**26**]. 

In vernal conjunctivitis, the histopathological examination of the conjunctiva shows epithelial hyperplasia, proliferation of fibrovascular connective tissue, and infiltrate with mast cells, eosinophils, lymphocytes, plasma cells, and monocytes. The scrapings of the superior tarsal conjunctiva show eosinophiles and free eosinophilic granules. 

In atopic keratoconjunctivitis, the histopathological examination may show an infiltrate of mast cells and eosinophils, epithelial pseudo gland formation, and enhanced number of goblet cells. In substantia propria, mast cells and mononuclear cell infiltration can be found. Conjunctival cytology may reveal eosinophils but not as abundant as in vernal keratoconjunctivitis, and not degranulated.

In giant papillary conjunctivitis, histology of the conjunctiva is distinguished by thick and irregular epithelium with prominent pseudo glands of Henle, decreased density of goblet cells in the apices of the papillae and increased in the crypts. Mast cells, eosinophils and basophils are raised in number in the epithelium and in the substantia propria. High concentration of immunoglobulins (IgM, IgE, IgG) may be found in the tears. 

The histologic exam of the nasal mucosa in allergic rhinitis has special features, presenting with nasal mucosal oedema, vasodilatation, glandular hyperplasia, and eosinophils infiltration in the lamina propria [**27**]. The nasal smear for eosinophils is considered a reliable diagnostic test for allergic rhinitis with moderately high sensitivity and high specificity [**28**,**29**].

## Diagnosis

The diagnosis of rhinoconjunctivitis is established by the concordance between the symptoms and the diagnostic tests. The simultaneity of the rhinitis with the ocular manifestations is of paramount importance. The symptoms encountered in the rhinoconjunctivitis are rhinorrhea, nasal obstruction, sneezing, nasal itching, tearing, swelling, ocular itching. There are special characteristics of the face of the patient with allergic conjunctivitis. The nasal crease is a horizontal crease on the nose bridge caused by the rubbing of the nose (allergic salute). Allergic shiners are dark circles around the eyes explained by the vasodilatation and congestion. The rhinoscopy alone cannot distinguish between the allergic and nonallergic types of rhinitis. Usually, a pale, swollen, bluish-gray mucosa in considered to be typical for allergy. The secretions typical to allergic rhinitis are watery and thin [**30**].

In perennial and seasonal allergic conjunctivitis, ocular examination may reveal classic signs such as oedema and congestion of the conjunctiva with excess of tearing. The conjunctiva often has a milky appearance due to obscuration of the vessels in substantia propria by oedema. Eyelid oedema may be present and sometimes prominent creases below the inferior eyelid can be noticed. 

The diagnostic tests should reveal the existence of an allergen specific IgE in vivo or in vitro. The gold standard is the skin-prick test. Detection of IgE to allergens is considered a second-line test [**31**]. Sometimes, in pollen allergies, differences between the results of the two tests can be observed. This phenomenon is explained by pan allergenic sensitization [**32**].

## Management

The therapy of allergic rhinoconjunctivitis is based on patient education, pharmacotherapy, and allergen-specific immunotherapy [**1**,**33**].

Pharmacotherapy is based on local intranasal corticosteroids. Studies have shown that intranasal corticosteroids are effective not only in controlling the nasal symptoms, but also the ocular symptoms [**34**]. High local concentrations of the active principle can be attained in the nasal mucosa without systemic adverse effects. Local corticosteroids administered locally are usually well tolerated with only minor side effects. There are no major differences between the efficacy of different corticosteroid preparations. Studies have shown that their efficacity is quite similar [**35**]. New intranasal corticosteroids are also effective on the ocular allergic symptoms, showing efficacy through the suppression of the nasal-ocular reflex, down regulating the inflammatory cell expression, and re-establishing the patency of the nasolacrimal duct [**36**].

Antihistamines are also used in the therapy of rhinoconjunctivitis. The mechanism of action of antihistamines is the interference with the histamine receptors H1 in the nasal and ocular mucosae. Second generation antihistamines have a higher specificity for the H1 receptors and do not cross the blood-brain barrier and therefore they are favored, causing less sedation [**37**]. 

Topical decongestants and corticosteroids are used to control the ocular symptoms, but the safety and optimal dosing regimen is still a matter of debate. Topical antihistamines and mast-cell stabilizers or double action drugs are the first line of treatment [**38**]. Ophthalmic lubricants (artificial tears or ointments) can be utilized to increase the humidity of the eye.

Allergic specific immunotherapy is nowadays recognized as an effective therapy for allergic diseases. It is recommended in allergic rhinitis and allergic conjunctivitis irrespective of their association with asthma, but when there is evidence of specific IgE sensitization for a relevant inhalant allergen [**39**].

## Conclusion

Allergic rhinoconjunctivitis, the allergic inflammatory disease of the nasal mucosa and of the ocular conjunctiva, is a condition encountered with an increased frequency, related to an important decrease of the quality of life. The conjunctiva and the nasal mucosa have the same type of epithelium, this explaining why the reactivity to both allergens is similar. The allergic pathophysiological mechanisms involved in allergic rhinitis and conjunctivitis also share common features. The anatomic connection through the nasolacrimal duct, the nasal-ocular reflex, and the systemic inflammatory response, explain the relationship between the allergic rhinitis and conjunctivitis and the concomitance of the manifestations. The diagnosis of rhinoconjunctivitis is based on the concordance between the symptoms, the clinical examination, and the diagnostic tests that should reveal the existence of an allergen specific IgE in vivo or in vitro. Whilst the nasal smear for eosinophils is considered a reliable diagnostic test for allergic rhinitis, the occurrence of eosinophils in the conjunctive is not a reliable indicator of allergy. The therapy of allergic rhinoconjunctivitis is based on patient education, pharmacotherapy, and allergen-specific immunotherapy. The local treatment of allergic rhinitis is mainly based on topical corticosteroids that also control the ocular symptoms. The first line of treatment of the ocular manifestations is represented by topical antihistamines and mast-cell stabilizers or double action drugs.

**Conflict of Interest statement**

The authors state no conflict of interest.

**Acknowledgements**

None.

**Sources of Funding**

None.

**Disclosures**

None.

## References

[1] del Giudice MM (2020). Allergic rhinoconjunctivitis. Acta Biomedica.

[2] Schatz M (2007). A survey of the burden of allergic rhinitis in the USA. Allergy.

[3] Canonica GW, Bousquet J, Mullol J, Scadding GK, Virchow JC (2007). A survey of the burden of allergic rhinitis in Europe. Allergy.

[4] Blaiss MS, Hammerby E, Robinson S, Kennedy-Martin T, Buchs S (2018). The burden of allergic rhinitis and allergic rhinoconjunctivitis on adolescents. Annals of Allergy, Asthma & Immunology.

[5] Nathan RA (2007). The burden of allergic rhinitis. Allergy and Asthma Proceedings.

[6] Bousquet J (2005). Characteristics of intermittent and persistent allergic rhinitis: DREAMS study group. Clinical Experimental Allergy.

[7] Kosina-Hagyó K (2012). Tear Film Function in Patients with Seasonal Allergic Conjunctivitis Outside the Pollen Season. International Archives of Allergy and Immunology.

[8] Bonini S, Coassin M, Aronni S, Lambiase A (2004). Vernal keratoconjunctivitis. Eye.

[9] Hogan MJ (1953). Atopic Keratoconjunctivitis. American Journal of Ophthalmology.

[10] Elhers WH, Donshik PC (2008). Giant papillary conjunctivitis. Current Opinion in Allergy & Clinical Immunology.

[11] Singh K, Axelrod S, Bielory L (2010). The epidemiology of ocular and nasal allergy in the United States, 1988-1994. Journal of Allergy and Clinical Immunology.

[12] Berger W (2005). Effects of adjuvant therapy with 0.1% olopatadine hydrochloride ophthalmic solution on quality of life in patients with allergic rhinitis using systemic or nasal therapy. Annals of Allergy, Asthma & Immunology.

[13] Bousquet J (1993). Heterogeneity of atopy. I. Clinical and immunologic characteristics of patients allergic to cypress pollen. Allergy.

[14] Scadding GK, Richards DH, Price MJ (2000). Patient and physician perspectives on the impact and management of perennial and seasonal allergic rhinitis. Clinical Otolaryngology and Allied Sciences.

[15] Dispenza MC (2019). Classification of hypersensitivity reactions. Allergy and Asthma Proceedings.

[16] Uzzaman A, Cho SH (2012). Chapter 28: Classification of hypersensitivity reactions. Allergy and Asthma Proceedings.

[17] Hansen I, Klimek L, Mösges R, Hörmann K (2004). Mediators of inflammation in the early and the late phase of allergic rhinitis. Current Opinion in Allergy and Clinical Immunology.

[18] Rosenwasser LJ (2011). Current Understanding of the Pathophysiology of Allergic Rhinitis. Immunology and Allergy Clinics of North America.

[19] Giavina-Bianchi P, Aun M, Takejima P, Kalil J, Agondi R (2016). United airway disease: current perspectives. Journal of Asthma and Allergy.

[20] Hom MM, Bielory L (2013). The Anatomical and Functional Relationship between Allergic Conjunctivitis and Allergic Rhinitis. Allergy & Rhinology.

[21] Sanke R (1989). Pseudonasolacrimal duct obstruction caused by nasal allergy. Ophthalmic Surg.

[22] Bernstein DI (2004). Treatment with intranasal fluticasone propionate significantly improves ocular symptoms in patients with seasonal allergic rhinitis. Clinical Experimental Allergy.

[23] Baroody FM, Naclerio RM (2011). Nasal-Ocular Reflexes and Their Role in the Management of Allergic Rhinoconjunctivitis With Intranasal Steroids. World Allergy Organization Journal.

[24] Li J (2005). Haemopoietic mechanisms in murine allergic upper and lower airway inflammation. Immunology.

[25] Saini S (2004). Systemic effects of allergen exposure on blood basophil IL-13 secretion and FcεRIβ. BJournal of Allergy and Clinical Immunology.

[26] Kari O (2009). Atopic conjunctivitis. Acta Ophthalmologica.

[27] Xu Y-Y, Liu X, Dai L-B, Zhou S-H (2012). Effect of Tong Qiao drops on the expression of exotoxin, IL-13 in the nasal mucosa of rats with allergic rhinitis. Journal of the Chinese Medical Association.

[28] Ahmadiafshar A, Taghiloo D, Esmailzadeh A, Falakaflaki B (2012). Nasal Eosinophilia as a Marker for Allergic Rhinitis: A Controlled study of 50 Patients. Ear, Nose & Throat Journal.

[29] Miller RE (1982). The Nasal Smear for Eosinophils. American Journal of Diseases of Children.

[30] Binder E, Holopainen E, Malmberg H, Salo O (1984). Clinical findings in patients with allergic rhinitis. Rhinology.

[31] Cipriani F (2018). Diagnostic relevance of IgE sensitization profiles to eight recombinant Phleum pratense molecules. Allergy.

[32] Mastrorilli C, Posa D, Cipriani F, Caffarelli C (2016). Asthma and allergic rhinitis in childhood: what’s new. Pediatric Allergy and Immunology.

[33] Licari A (2014). Current recommendations and emerging options for the treatment of allergic rhinitis. Expert Review of Clinical Immunology.

[34] Ratner P (2015). Efficacy of daily intranasal fluticasone propionate on ocular symptoms associated with seasonal allergic rhinitis. Annals of Allergy, Asthma & Immunology.

[35] Fowler J, Rotenberg BW, Sowerby LJ (2021). The subtle nuances of intranasal corticosteroids. Journal of Otolaryngology - Head & Neck Surgery.

[36] Bielory L (2010). Allergic Conjunctivitis and the Impact of Allergic Rhinitis. Current Allergy and Asthma Reports.

[37] Riechelmann H (2005). Orale Antihistaminika der 2. Generation bei allergischer Rhinitis. Laryngo-Rhino-Otologie.

[38] Leonardi A (2019). Management of ocular allergy. Allergy.

[39] Pajno GB (2017). Clinical practice recommendations for allergen-specific immunotherapy in children: the Italian consensus report. Italian Journal of Pediatrics.

